# Heart Failure after Aortic Valve Replacement: Incidence, Risk Factors, and Implications

**DOI:** 10.3390/jcm12186048

**Published:** 2023-09-19

**Authors:** Roopesh Sai Jakulla, Satya Preetham Gunta, Chetan P. Huded

**Affiliations:** 1 Department of Internal Medicine, University of Missouri-Kansas City, Kansas City, MO 64110, USA; 2 Department of Cardiology, Medical College of Wisconsin, Milwaukee, WI 53226, USA; 3 Department of Cardiology, University of Missouri-Kansas City, Kansas City, MO 64110, USA

**Keywords:** aortic stenosis, aortic valve replacement, heart failure, Kansas City cardiomyopathy questionnaire, transcatheter aortic valve replacement, surgical aortic valve replacement

## Abstract

Severe aortic stenosis (AS) carries a poor prognosis with the onset of heart failure (HF) symptoms, and surgical or transcatheter aortic valve replacement (AVR) is its only definitive treatment. The management of AS has seen a paradigm shift with the adoption of transcatheter aortic valve replacement (TAVR), allowing for the treatment of AS in patients who would not otherwise be candidates for surgical AVR. Despite improving long-term survival after TAVR in recent years, residual HF symptoms and HF hospitalization are common and are associated with an increased mortality and a poor health status. This review article summarizes the incidence and risk factors for HF after AVR. Strategies for preventing and better managing HF after AVR are necessary to improve outcomes in this patient population. Extensive research is underway to assess whether earlier timing for AVR, prior to the development of severe symptomatic AS and associated extra-valvular cardiac damage, can improve post-AVR patient outcomes.

## 1. Introduction

Valvular heart disease represents an important global health problem, and its prevalence is expected to rise due to the aging population. Aortic stenosis (AS) is the most common type of valvular heart disease in the western world, and deaths related to it have been on the rise for the last two decades [1,2]. Degenerative or calcific AS results from progressive valve thickening and calcification [3], which ultimately lead to outflow tract obstruction of the left ventricle (LV). Left untreated, severe AS leads to progressive heart failure and death. Aortic valve replacement (AVR) prolongs survival and is the only definitive therapy for severe symptomatic AS [4]. Calcific AS was associated with an estimated 151,000 global deaths in 2021 and a loss of over 2 million disability-adjusted life years [5]. The classic symptoms of AS are angina, syncope, and dyspnea. Dyspnea is an ominous sign signaling the onset of heart failure (HF) and shift from a compensated to a decompensated phase in the progression of AS.

AVR, the only definitive therapy for severe AS, improves survival and health status. Current guidelines recommend AVR for patients with severe symptomatic AS [6,7]. Among asymptomatic patients, AVR is indicated in those with severe AS with an LV ejection fraction of <50%, and in those with very high aortic valve gradients (mean gradient > 60 mmHg) and a low operative risk [6,7]. Both surgical AVR (SAVR) and transcatheter AVR (TAVR) are established therapies for AS. The decision between TAVR and SAVR depends on the patient’s age, frailty, comorbidities, and valve anatomy. While younger patients with a low operative risk may be preferentially treated with SAVR, older patients with comorbidities that increase their surgical risk, such as prior chest radiation or porcelain aorta, may be preferentially treated with TAVR [8]. Multiple randomized controlled trials have established the role of TAVR in high-, intermediate-, and low-risk surgical candidates [9,10,11]. The number of TAVRs performed yearly is on the rise in the US and exceeded the number of SAVRs performed in 2019 [12].

Although AVR is necessary to treat severe symptomatic AS, there is increasing recognition that many patients continue to suffer from HF symptoms, HF hospitalization, and a poor health status despite successful AVR. While patients with a reduced LV ejection fraction due to AS may have recovery of their LV ejection fraction after AVR, HF symptoms can persist due to myocardial hypertrophy and fibrosis despite improved systolic function. Heart failure is the most common cause of hospitalization following TAVR, highlighting the prevalence and clinical impact of this problem. Heart failure hospitalization after AVR is also associated with an increased risk of mortality [13,14,15,16,17].

## 2. Case Example of HF after Successful AVR

An 80-year-old woman with a low-risk Society of Thoracic Surgeons (STS) predictive risk of operative mortality of 2.6% was referred for AVR. Pre-AVR evaluation revealed an LV ejection fraction of 75%, mean aortic valve gradient of 40 mmHg consistent with severe AS, mild mitral and tricuspid valve regurgitation, normal right ventricular size and function, and an estimated pulmonary artery systolic pressure of 73 mmHg. Cardiac catheterization showed no obstructive coronary artery disease. She was treated with TAVR using a self-expanding transcatheter heart valve, with a successful procedure. Her post-procedure mean aortic valve gradient was 5 mmHg, with an LV ejection fraction of 70% and no paravalvular leak. Post-procedure complete heart block developed, which required the implantation of a dual-chamber permanent pacemaker. She developed new-onset atrial fibrillation early after TAVR treated with cardioversion. Despite a technically successful TAVR procedure, the patient suffered from 2 HF hospitalizations within 1 year post-TAVR and died 18 months post-TAVR. This case highlights a common clinical scenario in the current era: a patient with severe symptomatic AS treated with successful TAVR who continues to struggle with clinically significant HF, leading to death relatively early after TAVR.

## 3. Incidence of HF Hospitalization after AVR

**Figure 1:**
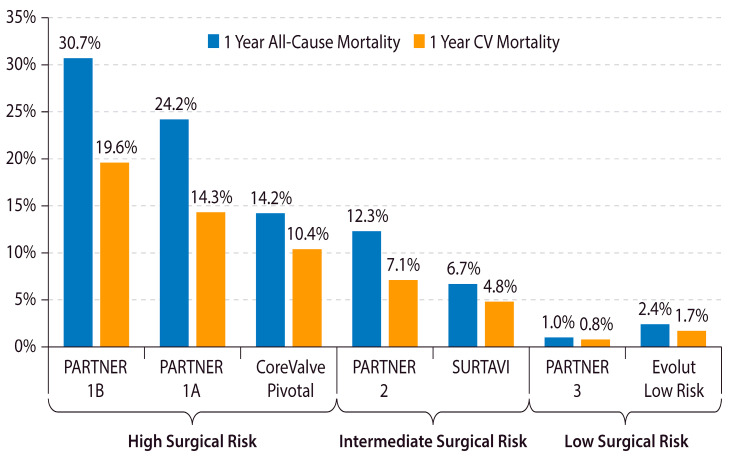
Declining 1-year mortality after TAVR in lower-risk Patients. The 1-year all-cause and cardiovascular mortality after TAVR in major TAVR clinical trials is shown. CV = cardiovascular. PARTNER = Placement of Aortic Transcatheter Valves. SURTAVI = Surgical Replacement and Transcatheter Aortic Valve Implantation [9,10,11,19,20,21,22].

The outcomes of TAVR have improved over time with device improvements, refined procedural techniques, and lower-risk patient populations. A study from the Transcatheter Valve Therapy (TVT) registry demonstrated that, among 12,182 patients treated with TAVR in the United States between 2011 and 2013, the rate of HF readmission at 1 year was 14.3% and the 1-year overall mortality was 23.7% [18]. However, 1-year overall mortality has decreased substantially in recent years and is now 10% in clinical practice and <2% in recent clinical trials on low-risk patients (Figure 1) [9,10,11,12,19,20,21,22].

Despite these improvements in survival post-TAVR, the incidence of HF hospitalization after TAVR remains a concern. The incidence of HF after TAVR reported in various registries and clinical trials ranges from 7 to 24% [14,16,18,23,24,25,26] (Table 1). This is comparable to the incidence of HF hospitalization in clinical trials on chronic systolic heart failure patients [27,28]. The HF rate post-AVR is higher in observational studies from registries compared to clinical trials. The difference between these trial and real-world practice HF rates may be due to the frequency of follow-ups, the intensity of medical therapy, or the Hawthorne effect, where study participants have lower rates as a result of being observed in a trial setting [29]. In the TVT Registry, HF was the most common reason for readmission within the first year after TAVR [26]. A recent post hoc analysis of 3403 TAVR and SAVR patients included in the PARTNER (Placement of Aortic Transcatheter Valves) I, II, and III trials demonstrated that HF hospitalizations within 1 year after AVR are associated with an increased mortality and worse 1-year health status, irrespective of the type of AVR (TAVR or SAVR) [14].

## 4. The Kansas City Cardiomyopathy Questionnaire: A Tool to Evaluate HF Symptoms

The aim of AVR is to improve both longevity and health status (symptoms, physical functioning, and quality of life). Changes in health status after AVR can be accurately and reliably measured using the Kansas City Cardiomyopathy Questionnaire (KCCQ). The KCCQ was originally developed as a tool for measuring the symptoms, social and physical limitations, quality of life, and self-efficacy in HF patients and has been validated as a reliable instrument for measuring the health status of severe AS patients [32,33]. It is a 23-item self-administered questionnaire that measures 7 specific health domains relevant to HF. The domain scores and 3 summary scores are each represented on a 0–100 scale, with higher scores indicating a better health status [34]. An abbreviated 12-item version, the KCCQ-12, has been validated and has an excellent concordance with the original 23-item scale. The KCCQ overall summary score (KCCQ-OS) is commonly reported as a summary assessment of the health status of AS patients before and after AVR. To improve their interpretability, KCCQ-OS scores are often analyzed in 25-point ranges (0–24, 25–49, 50–74, and 75–100), representing very poor to poor, poor to fair, fair to good, and good to excellent health statuses [34].

**Figure 2:**
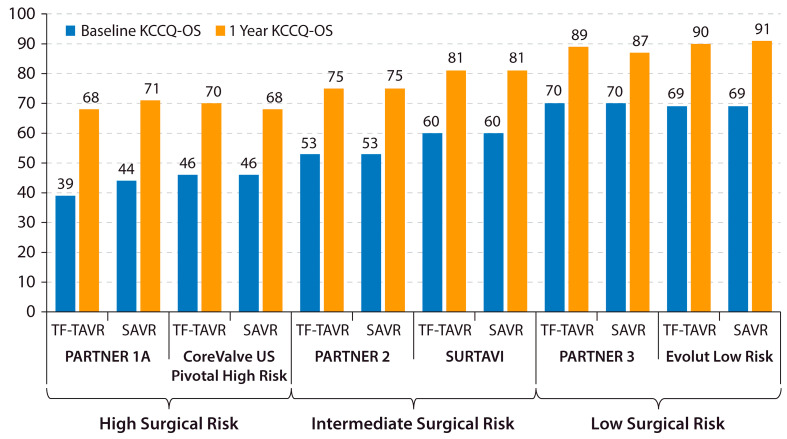
Changes in KCCQ-OS after AVR in major trials. Baseline and 1-year KCCQ-OS is shown for the TF-TAVR and SAVR arms of recent major AVR trials across the surgical risk spectrum. KCCQ-OS = Kansas City Cardiomyopathy Questionnaire overall summary score. PARTNER = Placement of Aortic Transcatheter Valves. SAVR = surgical aortic valve replacement. SURTAVI = Surgical Replacement and Transcatheter Aortic Valve Implantation. TF-TAVR = transfemoral transcatheter aortic valve replacement. US = United States [19,36,37,38,39].

Both TAVR and SAVR have shown remarkable improvements in KCCQ-OS from baseline to 1 year in recent clinical trials (Figure 2). Despite a large average treatment effect of AVR being evidenced by these clinical trial results, a substantial proportion of patients continue to remain symptomatic with HF after AVR. This discrepancy highlights the importance of identifying patients with residual HF symptoms despite a successful AVR procedure. A major advance in this area was the development of a “poor outcome” after TAVR, a concept that was introduced by Arnold et al. in 2013 [35]. In the current era, a “poor outcome” after TAVR is defined as death, KCCQ-OS of <60, or a decline in KCCQ-OS by 10 or more points from baseline to 1-year post-TAVR. This framework allows investigators to identify patients who either did not survive or are living with a low or declining health status 1 year after AVR.

Remarkably, in practice, a large proportion of patients meet the definition of a poor outcome 1 year after TAVR, and, although this is declining [40], the decline in poor outcomes is far less than the rate of decline in 1-year post-TAVR mortality. In 2018, the rate of poor outcomes after TAVR was 32%, with 19% of patients having a low or declining health status [12]. These data highlight that, even in the modern era of TAVR, 1 out of 3 patients die or suffer from a substantial HF symptom burden with a low or declining health status 1 year after TAVR. This observation underscores the need for strategies to better identify and treat these patients longitudinally pre- and post-AVR.

## 5. Risk Factors for Poor Outcome and HF after AVR

Various risk models have been developed to predict patients at risk of poor outcomes based on pre-procedure patient characteristics [41]. Among the high-risk patients treated in PARTNER I, predictors of a poor outcome at 1 year included a higher baseline creatinine level, oxygen-dependent lung disease, a lower baseline mean aortic valve gradient, a lower baseline score on the Mini-Mental Status Examination, and a shorter baseline 6 min walk test distance [41]. An updated model from the TVT Registry reported a lower baseline KCCQ-OS, a lower mean aortic valve gradient, home oxygen use, a higher baseline creatinine level, atrial fibrillation or flutter, and diabetes mellitus as significant predictors of 1-year poor outcomes [40]. A similar model was developed among the high-risk patients treated in the CoreValve US Pivotal Extreme and High-Risk trials of self-expanding TAVR devices, which identified the same key clinical variables (KCCQ-OS, mean aortic valve gradient, home oxygen, creatinine level, cognitive function, atrial fibrillation or flutter, and diabetes), but further added frailty as a component [42]. These risk models highlight that poor outcomes after AVR, either with death or a low health status, are associated with major non-cardiac comorbidities such as chronic lung disease, renal dysfunction, diabetes, and frailty.

Risk factors for HF hospitalization after TAVR have also been studied and are largely similar to the factors associated with the poor outcome metric (Table 2). A recent analysis of 3403 pooled TAVR and SAVR patients across the low-, intermediate-, and high-risk cohorts from the PARTNER trials identified a low baseline aortic valve mean gradient, atrial fibrillation or flutter, and prior coronary revascularization as factors associated with 1-year HF hospitalization [14]. Several prior registry studies have identified atrial fibrillation, renal insufficiency, a lower aortic valve mean gradient, higher surgical risk scores, diabetes mellitus, and a lower LV ejection fraction as risk factors for HF hospitalization after TAVR [16,23,24,25,43,44,45]. In addition to baseline patient characteristics, several post-procedure issues may also increase the risk of HF symptoms and hospitalization. Paravalvular regurgitation [46], patient–prosthesis mismatch [47,48], new-onset left bundle branch block, and the need for permanent pacemaker implantation [49,50] are associated with an increased incidence of HF hospitalization after TAVR.

There is a paucity of contemporary data evaluating the risks for HF hospitalization after SAVR. However, the aforementioned analysis of the pooled TAVR and SAVR patients treated in the PARTNER trials found that treatment type (TAVR vs. SAVR) was not associated with the risk of HF hospitalization within 1 year after AVR [14]. This finding suggests that the risk of HF hospitalization is similar between AVR types. Additionally, treatment type (TAVR vs. SAVR) was not an effect modifier on the relationship between post-AVR HF hospitalization and poor 1-year outcomes, such as death or a low health status. This finding indicates that patients did similarly poorly in terms of HF after AVR, regardless of whether their AVR type was TAVR or SAVR.

In addition to pre-procedural patient characteristics and post-procedural valve-related complications, post-procedure health status has also been shown to have important prognostic implications for patients undergoing transcatheter valve procedures. A recent study of 67,669 patients treated with either TAVR or mitral transcatheter edge-to-edge repair from the TVT registry demonstrated that both baseline and 30-day KCCQ scores were predictive of HF hospitalization and death, with the 30-day assessment having the strongest association with 1-year outcomes [51]. These results point to the utility of the 30-day post-procedure assessment to identify high-risk patients for poor outcomes and implement novel strategies for managing post-TAVR HF before poor outcomes ensue.

## 6. HF Hospitalization as a Clinical Trial Endpoint for Aortic Stenosis

The Valve Academic Research Consortium (VARC) was founded in 2010 as a multidisciplinary collaboration with the goal of creating consistent endpoint definitions for both surgical and transcatheter aortic valve clinical research [52]. In the last decade, major HF trials have used HF hospitalizations or rehospitalizations as an important clinical endpoint due to the association of rehospitalization events with poor prognoses [27,53,54]. More recently, rehospitalization endpoints have emerged as a key component of trials on AS. Published in 2021, the VARC-3 updated these endpoint definitions to support the use of HF hospitalization as a specific endpoint in AVR trials [55]. The PARTNER 3 trial, which is the most recent in the PARTNER series, was the first AVR trial to incorporate rehospitalizations as a component of its primary endpoint (Table 3) [10]. Although HF hospitalizations have been adopted in transcatheter valve trials, the challenges that accompany them must be acknowledged. For example, the thresholds for hospitalization for HF may vary due to local practice patterns and social or geographical factors, which are difficult to assess. Despite these challenges, a recent study from the PARTNER trial demonstrated the independent association of HF rehospitalizations, after both TAVR and SAVR, with poor outcomes, mortality, and a substantial decline in health status 1 year after AVR [14]. These findings support the continued use of HF hospitalization as a key clinical outcome after AVR.

## 7. The Role of Early AVR to Mitigate HF after AVR

AS is characterized by progressive valve narrowing and consequent concentric left ventricular hypertrophy (LVH), making it a disease of both the aortic valve and the myocardium. LVH is a result of ventricular wall stress and pressure overload on the LV. The severity of stenosis and degree of myocardial hypertrophic response determine the rate of AS progression and the need for AVR [56]. The adaptive hypertrophic response transitions to maladaptive cardiomyopathy and ultimately to decompensated heart failure. An increased LV mass is also associated with an increased rate of HF and cardiac events in patients with asymptomatic severe AS [57].

Given the established natural history of AS as a progressive condition with associated myocardial disease, Genereux et al. proposed a cardiac damage hypothesis and developed a staging system for AS based on extra-valvular cardiac damage detected via echocardiogram prior to AVR [58]. Severe AS patients from PARTNER 2 trials were pooled and classified into five stages: no cardiac damage (stage 0), LV damage (stage 1), left atrial or mitral valve damage (stage 2), pulmonary vasculature or tricuspid damage (stage 3), and right ventricular damage (stage 4). The study demonstrated a significantly increased cardiac mortality with an increment in each stage, suggesting the need to revisit the optimal timing of AVR, ideally prior to the onset of progressive extra-valvular damage. Further work among the patients pooled from the PARTNER 2 and 3 trials has shown that each 1-stage increase in baseline cardiac damage is associated with a 24% increase in the odds of a poor outcome 1-year post-AVR [59].

The optimal timing of AVR in asymptomatic AS patients remains a controversial topic. The European and US guidelines recommend AVR for asymptomatic AS patients with a low LV ejection fraction (<50%), low-surgical-risk patients with abnormal exercise testing, very severe AS (aortic velocity of >5 m/s), elevated serum brain natriuretic peptide (BNP), and an increase in aortic velocity of 0.3 m/s every year on serial imaging [6,7]. The US guidelines additionally note a progressive decrease in LV ejection fraction on at least three serial studies to <60%. However, the level of evidence behind these indications is low and based on expert opinion or small, retrospective, and observational studies. Multiple meta-analyses of observational trials including asymptomatic severe AS patients suggest a benefit of early AVR compared to a conventional surveillance strategy [60,61,62,63].

Two recent randomized controlled trials of SAVR vs. surveillance provided important insights. In the RECOVERY trial, 145 patients with a mean aortic valve gradient of ≥50 mmHg or peak aortic valve velocity of >4.5 m/s were randomly assigned to early surgery or conservative care. Patients in the early SAVR group had less operative and cardiovascular mortality during follow-up compared to the conservative group [64]. In that study, there was a 67% relative risk reduction and 22% absolute risk reduction in all-cause mortality over 8 years of follow-up. The results should be interpreted with caution, as the patients were relatively young with few comorbidities and low operative risks. The AVATAR trial was the second RCT with results in favor of early SAVR in severe AS patients, even before symptom onset [65]. In AVATAR, 157 patients with a mean aortic valve gradient of ≥ 40 mmHg were followed for 4 years, and the early SAVR arm had a significantly lower risk of the primary composite outcomes of death, acute myocardial infarction, stroke, or HF hospitalization (15.2% vs. 34.7%, *p* = 0.02). HF hospitalization was at 4.0% in the early SAVR group compared to 12.9% in the surveillance group. These findings are intriguing, especially when a less invasive option like TAVR is available. In this context, multiple RCTs are underway to evaluate whether early TAVR is beneficial compared to expectant management in asymptomatic severe AS-EARLY TAVR (NCT03042104), EASY-AS (NCT04204915), and EVoLVeD (NCT03094143).

## 8. Conclusions

AVR improves long-term survival and health status among patients with severe symptomatic AS. Despite major improvements in 1-year post-TAVR survival over the past decade, many patients continue to suffer from HF hospitalizations and poor health status. Health status is an important aspect of AS management and powerful tools such as the KCCQ can be clinically leveraged to guide HF management after AVR. Due to its association with mortality, HF hospitalization has emerged as a clinical endpoint in AS trials. Although risk factors have been identified that predict HF and poor outcomes after AVR, many such risk factors are non-modifiable, and withholding AVR based on these factors does not appear justified. The current focus is shifting toward early intervention for AS, prior to the onset of cardiac damage or clinical symptoms, as a potential strategy for improving outcomes after AVR.

## Figures and Tables

**Table 1:** Incidence of heart failure hospitalization after aortic valve replacement.

Publication Year	Author	N	AVR Type	Design	1-Year HF Hospitalization
2022	Huded et al. [14]	3403	SAVR and TAVR	Secondary analysis of RCTs	6.7%
2020	Auffret et al. [30]	808	TAVR	Single-center retrospective	13.6%
2019	Vemulapalli et al. [26]	15,324	TAVR	Multicenter retrospective	14.2%
2019	Guedeney et al. [16]	1139	TAVR	Multicenter prospective	9.2%
2019	Harbaoui et al. [24]	409	TAVR	Multicenter prospective	19.9%
2018	Nazzari et al. [25]	742	TAVR	Multicenter prospective	12.4%
2017	Durand et al. [23]	546	TAVR	Single-center retrospective	24.1%
2017	Forcillo et al. [31]	714	TAVR	Single-center retrospective	7.6%
2015	Holmes Jr et al. [18]	12,182	TAVR	Multicenter retrospective	14.3%

AVR = aortic valve replacement. HF = heart failure. RCT = randomized controlled trial. SAVR = surgical aortic valve replacement. TAVR = transcatheter aortic valve replacement.

**Table 2:** Established risk factors for HF hospitalization or poor outcome after AVR.

Baseline Characteristics	Post-Procedural Characteristics
Lower baseline KCCQ-OS	Paravalvular regurgitation
Lower baseline aortic valve mean gradient	Patient–prosthesis mismatch
Higher baseline creatinine	New left bundle branch block
Atrial fibrillation or flutter	New permanent pacemaker
Diabetes mellitus	Lower 30-day KCCQ-OS
Oxygen-dependent lung disease	
Frailty	
Poor cognitive function	

Poor outcome defined as death, KCCQ-OS < 60, or decline of ≥ 10 points from baseline after AVR. AVR = aortic valve replacement. HF = heart failure. KCCQ = Kansas City Cardiomyopathy Questionnaire overall summary score.

**Table 3:** Changes in the primary outcome of balloon expandable TAVR trials.

Surgical Risk	Year	Trial	Primary Outcome
Extreme	2010	PARTNER 1B [21]	Death
High	2011	PARTNER 1A [11]	Death
Intermediate	2016	PARTNER 2 [9]	Death or disabling stroke
Low	2019	PARTNER 3 [10]	Death, stroke, or rehospitalization due to the procedure, valve, or heart failure

PARTNER = placement of aortic transcatheter valves. TAVR = transcatheter aortic valve replacement.

## Data Availability

Not applicable.

## Abbreviations

AS	Aortic Stenosis
AVR	Aortic Valve Replacement
HF	Heart Failure
KCCQ	Kansas City Cardiomyopathy Questionnaire
KCCQ-OS	Kansas City Cardiomyopathy Questionnaire Overall summary Score
LV	Left Ventricle
PARTNER	Placement of Aortic Transcatheter Valves
SAVR	Surgical Aortic Valve Replacement
STS	Society of Thoracic Surgeons
TAVR	Transcatheter Aortic Valve Replacement
TVT	Transcatheter Valve Therapy
VARC	Valve Academic Research Consortium

## References

[1] Coffey S., Roberts-Thomson R., Brown A., Carapetis J., Chen M., Enriquez-Sarano M., Zühlke L., Prendergast B.D. (2021). Global epidemiology of valvular heart disease. Nat. Rev. Cardiol..

[2] Tsao C.W., Aday A.W., Almarzooq Z.I., Alonso A., Beaton A.Z., Bittencourt M.S., Boehme A.K., Buxton A.E., Carson A.P., Commodore-Mensah Y. (2022). Heart Disease and Stroke Statistics-2022 Update: A Report From the American Heart Association. Circulation.

[3] Pawade T.A., Newby D.E., Dweck M.R. (2015). Calcification in Aortic Stenosis: The Skeleton Key. J. Am. Coll. Cardiol..

[4] Schwarz F., Baumann P., Manthey J., Hoffmann M., Schuler G., Mehmel H.C., Schmitz W., Kübler W. (1982). The effect of aortic valve replacement on survival. Circulation.

[5] Vaduganathan M., Mensah G.A., Turco J.V., Fuster V., Roth G.A. (2022). The Global Burden of Cardiovascular Diseases and Risk. J. Am. Coll. Cardiol..

[6] Otto C.M., Nishimura R.A., Bonow R.O., Carabello B.A., Erwin J.P., Gentile F., Jneid H., Krieger E.V., Mack M., McLeod C. (2021). 2020 ACC/AHA Guideline for the Management of Patients With Valvular Heart Disease: A Report of the American College of Cardiology/American Heart Association Joint Committee on Clinical Practice Guidelines. Circulation.

[7] Vahanian A., Beyersdorf F., Praz F., Milojevic M., Baldus S., Bauersachs J., Capodanno D., Conradi L., De Bonis M., De Paulis R. (2021). 2021 ESC/EACTS Guidelines for the management of valvular heart disease: Developed by the Task Force for the management of valvular heart disease of the European Society of Cardiology (ESC) and the European Association for Cardio-Thoracic Surgery (EACTS). Eur. Heart J..

[8] Plonska-Gosciniak E., Piotrowski G., Wojakowski W., Gosciniak P., Olszowska M., Lesiak M., Klotzka A., Grygier M., Deja M., Kasprzak J.D. (2023). Management of valvular heart disease in patients with cancer: Multidisciplinary team, cancer-therapy related cardiotoxicity, diagnosis, transcatheter intervention, and cardiac surgery. Expert opinion of the Association on Valvular Heart Disease, Association of Cardiovascular Interventions, and Working Group on Cardiac Surgery of the Polish Cardiac Society. Kardiol. Pol..

[9] Leon M.B., Smith C.R., Mack M.J., Makkar R.R., Svensson L.G., Kodali S.K., Thourani V.H., Tuzcu E.M., Miller D.C., Herrmann H.C. (2016). Transcatheter or Surgical Aortic-Valve Replacement in Intermediate-Risk Patients. N. Engl. J. Med..

[10] Mack M.J., Leon M.B., Thourani V.H., Makkar R., Kodali S.K., Russo M., Kapadia S.R., Malaisrie S.C., Cohen D.J., Pibarot P. (2019). Transcatheter Aortic-Valve Replacement with a Balloon-Expandable Valve in Low-Risk Patients. N. Engl. J. Med..

[11] Smith C.R., Leon M.B., Mack M.J., Miller D.C., Moses J.W., Svensson L.G., Tuzcu E.M., Webb J.G., Fontana G.P., Makkar R.R. (2011). Transcatheter versus Surgical Aortic-Valve Replacement in High-Risk Patients. N. Engl. J. Med..

[12] Carroll J.D., Mack M.J., Vemulapalli S., Herrmann H.C., Gleason T.G., Hanzel G., Deeb G.M., Thourani V.H., Cohen D.J., Desai N. (2020). STS-ACC TVT Registry of Transcatheter Aortic Valve Replacement. J. Am. Coll. Cardiol..

[13] Franzone A., Pilgrim T., Arnold N., Heg D., Langhammer B., Piccolo R., Roost E., Praz F., Räber L., Valgimigli M. (2017). Rates and predictors of hospital readmission after transcatheter aortic valve implantation. Eur. Heart J..

[14] Huded C.P., Arnold S.V., Chhatriwalla A.K., Saxon J.T., Kapadia S., Yu X., Webb J.G., Thourani V.H., Kodali S.K., Smith C.R. (2022). Rehospitalization Events After Aortic Valve Replacement: Insights From the PARTNER Trial. Circ. Cardiovasc. Interv..

[15] Zahid S., Din M.T.U., Khan M.Z., Rai D., Ullah W., Sanchez-Nadales A., Elkhapery A., Khan M.U., Goldsweig A.M., Singla A. (2022). Trends, Predictors, and Outcomes of 30-Day Readmission With Heart Failure After Transcatheter Aortic Valve Replacement: Insights From the US Nationwide Readmission Database. J. Am. Heart Assoc..

[16] Guedeney P., Huchet F., Manigold T., Rouanet S., Balagny P., Leprince P., Lebreton G., Letocart V., Barthelemy O., Vicaut E. (2019). Incidence of, risk factors for and impact of readmission for heart failure after successful transcatheter aortic valve implantation. Arch. Cardiovasc. Dis..

[17] Verheul H.A., Brink R.B.A.v.d., Bouma B.J., Hoedemaker G., Moulijn A.C., Dekker E., Bossuyt P., Dunning A.J. (1995). Analysis of risk factors for excess mortality after aortic valve replacement. J. Am. Coll. Cardiol..

[18] Holmes D.R., Brennan J.M., Rumsfeld J.S., Dai D., O’Brien S.M., Vemulapalli S., Edwards F.H., Carroll J., Shahian D., Grover F. (2015). Clinical Outcomes at 1 Year Following Transcatheter Aortic Valve Replacement. JAMA.

[19] Popma J.J., Deeb G.M., Yakubov S.J., Mumtaz M., Gada H., O’Hair D., Bajwa T., Heiser J.C., Merhi W., Kleiman N.S. (2019). Transcatheter Aortic-Valve Replacement with a Self-Expanding Valve in Low-Risk Patients. N. Engl. J. Med..

[20] Adams D.H., Popma J.J., Reardon M.J., Yakubov S.J., Coselli J.S., Deeb G.M., Gleason T.G., Buchbinder M., Hermiller J., Kleiman N.S. (2014). Transcatheter Aortic-Valve Replacement with a Self-Expanding Prosthesis. N. Engl. J. Med..

[21] Leon M.B., Smith C.R., Mack M., Miller D.C., Moses J.W., Svensson L.G., Tuzcu E.M., Webb J.G., Fontana G.P., Makkar R.R. (2010). Transcatheter aortic-valve implantation for aortic stenosis in patients who cannot undergo surgery. N. Engl. J. Med..

[22] Reardon M.J., Van Mieghem N.M., Popma J.J., Kleiman N.S., Søndergaard L., Mumtaz M., Adams D.H., Deeb G.M., Maini B., Gada H. (2017). Surgical or Transcatheter Aortic-Valve Replacement in Intermediate-Risk Patients. N. Engl. J. Med..

[23] Durand E., Doutriaux M., Bettinger N., Tron C., Fauvel C., Bauer F., Dacher J.-N., Bouhzam N., Litzler P.-Y., Cribier A. (2017). Incidence, Prognostic Impact, and Predictive Factors of Readmission for Heart Failure After Transcatheter Aortic Valve Replacement. JACC Cardiovasc. Interv..

[24] Harbaoui B., Durand E., Dupré M., Rabilloud M., Souteyrand G., Courand P.-Y., Boussel L., Lefevre T., Eltchaninoff H., Lantelme P. (2019). Significance of the CAPRI risk score to predict heart failure hospitalization post-TAVI: The CAPRI-*HF* study. Int. J. Cardiol..

[25] Nazzari H., Hawkins N.M., Ezekowitz J., Lauck S., Ding L., Polderman J., Yu M., Boone R.H., Cheung A., Ye J. (2019). The Relationship Between Heart-Failure Hospitalization and Mortality in Patients Receiving Transcatheter Aortic Valve Replacement. Can. J. Cardiol..

[26] Vemulapalli S., Dai D., Hammill B.G., Baron S.J., Cohen D.J., Mack M.J., Holmes D.R. (2019). Hospital Resource Utilization Before and After Transcatheter Aortic Valve Replacement: The STS/ACC TVT Registry. J. Am. Coll. Cardiol..

[27] McMurray J.J.V., Packer M., Desai A.S., Gong J., Lefkowitz M.P., Rizkala A.R., Rouleau J.L., Shi V.C., Solomon S.D., Swedberg K. (2014). Angiotensin-Neprilysin Inhibition versus Enalapril in Heart Failure. N. Engl. J. Med..

[28] Moss A.J., Hall W.J., Cannom D.S., Klein H., Brown M.W., Daubert J.P., Estes N.A.M., Foster E., Greenberg H., Higgins S.L. (2009). Cardiac-Resynchronization Therapy for the Prevention of Heart-Failure Events. N. Engl. J. Med..

[29] McCarney R., Warner J., Iliffe S., van Haselen R., Griffin M., Fisher P. (2007). The Hawthorne Effect: A randomised, controlled trial. BMC Med. Res. Methodol..

[30] Auffret V., Bakhti A., Leurent G., Bedossa M., Tomasi J., Soulami R.B., Verhoye J.-P., Donal E., Galli E., Loirat A. (2020). Determinants and Impact of Heart Failure Readmission Following Transcatheter Aortic Valve Replacement. Circ. Cardiovasc. Interv..

[31] Forcillo J., Condado J.F., Binongo J.N., Lasanajak Y., Caughron H., Babaliaros V., Devireddy C., Leshnower B., Guyton R.A., Block P.C. (2017). Readmission rates after transcatheter aortic valve replacement in high- and extreme-risk patients with severe aortic stenosis. J. Thorac. Cardiovasc. Surg..

[32] Green C.P., Porter C.B., Bresnahan D.R., Spertus J.A. (2000). Development and evaluation of the Kansas City Cardiomyopathy Questionnaire: A new health status measure for heart failure. J. Am. Coll. Cardiol..

[33] Arnold S.V., Spertus J.A., Lei Y., Allen K.B., Chhatriwalla A.K., Leon M.B., Smith C.R., Reynolds M.R., Webb J.G., Svensson L.G. (2013). Use of the Kansas City Cardiomyopathy Questionnaire for Monitoring Health Status in Patients with Aortic Stenosis. Circ. Heart Fail..

[34] Spertus J.A., Jones P.G., Sandhu A.T., Arnold S.V. (2020). Interpreting the Kansas City Cardiomyopathy Questionnaire in Clinical Trials and Clinical Care: JACC State-of-the-Art Review. J. Am. Coll. Cardiol..

[35] Arnold S.V., Spertus J.A., Lei Y., Green P., Kirtane A.J., Kapadia S., Thourani V.H., Herrmann H.C., Beohar N., Zajarias A. (2013). How to Define a Poor Outcome After Transcatheter Aortic Valve Replacement. Circ. Cardiovasc. Qual. Outcomes.

[36] Reynolds M.R., Magnuson E.A., Wang K., Thourani V.H., Williams M., Zajarias A., Rihal C.S., Brown D.L., Smith C.R., Leon M.B. (2012). Health-related quality of life after transcatheter or surgical aortic valve replacement in high-risk patients with severe aortic stenosis: Results from the PARTNER (Placement of AoRTic TraNscathetER Valve) Trial (Cohort A). J. Am. Coll. Cardiol..

[37] Arnold S.V., Reynolds M.R., Wang K., Magnuson E.A., Baron S.J., Chinnakondepalli K.M., Reardon M.J., Tadros P.N., Zorn G.L., Maini B. (2015). Health Status After Transcatheter or Surgical Aortic Valve Replacement in Patients With Severe Aortic Stenosis at Increased Surgical Risk: Results From the CoreValve US Pivotal Trial. JACC Cardiovasc. Interv..

[38] Baron S.J., Magnuson E.A., Lu M., Wang K., Chinnakondepalli K., Mack M., Thourani V.H., Kodali S., Makkar R., Herrmann H.C. (2019). Health Status After Transcatheter Versus Surgical Aortic Valve Replacement in Low-Risk Patients With Aortic Stenosis. J. Am. Coll. Cardiol..

[39] Tuttle M.K., Kiaii B., Van Mieghem N.M., Laham R.J., Deeb G.M., Windecker S., Chetcuti S., Yakubov S.J., Chawla A., Hockmuth D. (2022). Functional Status After Transcatheter and Surgical Aortic Valve Replacement: 2-Year Analysis From the SURTAVI Trial. JACC Cardiovasc. Interv..

[40] Arnold S.V., Cohen D.J., Dai D., Jones P.G., Li F., Thomas L., Baron S.J., Frankel N.Z., Strong S., Matsouaka R.A. (2018). Predicting Quality of Life at 1 Year After Transcatheter Aortic Valve Replacement in a Real-World Population. Circ. Cardiovasc. Qual. Outcomes.

[41] Arnold S.V., Reynolds M.R., Lei Y., Magnuson E.A., Kirtane A.J., Kodali S.K., Zajarias A., Thourani V.H., Green P., Rodés-Cabau J. (2014). Predictors of poor outcomes after transcatheter aortic valve replacement: Results from the PARTNER (Placement of Aortic Transcatheter Valve) trial. Circulation.

[42] Arnold S.V., Afilalo J., Spertus J.A., Tang Y., Baron S.J., Jones P.G., Reardon M.J., Yakubov S.J., Adams D.H., Cohen D.J. (2016). Prediction of Poor Outcome After Transcatheter Aortic Valve Replacement. J. Am. Coll. Cardiol..

[43] Hioki H., Watanabe Y., Kozuma K., Nara Y., Kawashima H., Nagura F., Nakashima M., Kataoka A., Yamamoto M., Naganuma T. (2017). Timing of Susceptibility to Mortality and Heart Failure in Patients With Preexisting Atrial Fibrillation After Transcatheter Aortic Valve Implantation. Am. J. Cardiol..

[44] Salaun E., Clavel M.A., Hahn R.T., Jaber W.A., Asch F.M., Rodriguez L., Weissman N.J., Gertz Z.M., Herrmann H.C., Dahou A. (2020). Outcome of Flow-Gradient Patterns of Aortic Stenosis After Aortic Valve Replacement: An Analysis of the PARTNER 2 Trial and Registry. Circ. Cardiovasc. Interv..

[45] Yoshijima N., Saito T., Inohara T., Anzai A., Tsuruta H., Shimizu H., Fukuda K., Naganuma T., Mizutani K., Yamawaki M. (2021). Predictors and clinical outcomes of poor symptomatic improvement after transcatheter aortic valve replacement. Open Heart.

[46] Pibarot P., Hahn R.T., Weissman N.J., Arsenault M., Beaudoin J., Bernier M., Dahou A., Khalique O.K., Asch F.M., Toubal O. (2017). Association of Paravalvular Regurgitation With 1-Year Outcomes After Transcatheter Aortic Valve Replacement With the SAPIEN 3 Valve. JAMA Cardiol..

[47] Herrmann H.C., Daneshvar S.A., Fonarow G.C., Stebbins A., Vemulapalli S., Desai N.D., Malenka D.J., Thourani V.H., Rymer J., Kosinski A.S. (2018). Prosthesis-Patient Mismatch in Patients Undergoing Transcatheter Aortic Valve Replacement: From the STS/ACC TVT Registry. J. Am. Coll. Cardiol..

[48] Fallon J.M., DeSimone J.P., Brennan J.M., O’Brien S., Thibault D.P., DiScipio A.W., Pibarot P., Jacobs J.P., Malenka D.J. (2018). The Incidence and Consequence of Prosthesis-Patient Mismatch After Surgical Aortic Valve Replacement. Ann. Thorac. Surg..

[49] Faroux L., Chen S., Muntané-Carol G., Regueiro A., Philippon F., Sondergaard L., Jørgensen T.H., Lopez-Aguilera J., Kodali S., Leon M. (2020). Clinical impact of conduction disturbances in transcatheter aortic valve replacement recipients: A systematic review and meta-analysis. Eur. Heart J..

[50] Chamandi C., Barbanti M., Munoz-Garcia A., Latib A., Nombela-Franco L., Gutiérrez-Ibanez E., Veiga-Fernandez G., Cheema A.N., Cruz-Gonzalez I., Serra V. (2018). Long-Term Outcomes in Patients With New Permanent Pacemaker Implantation Following Transcatheter Aortic Valve Replacement. JACC Cardiovasc. Interv..

[51] Hejjaji V., Cohen D.J., Carroll J.D., Li Z., Manandhar P., Vemulapalli S., Nelson A.J., Malik A.O., Mack M.J., Spertus J.A. (2021). Practical Application of Patient-Reported Health Status Measures for Transcatheter Valve Therapies. Circ. Cardiovasc. Qual. Outcomes.

[52] Leon M.B., Piazza N., Nikolsky E., Blackstone E.H., Cutlip D.E., Kappetein A.P., Krucoff M.W., Mack M., Mehran R., Miller C. (2011). Standardized endpoint definitions for Transcatheter Aortic Valve Implantation clinical trials: A consensus report from the Valve Academic Research Consortium. J. Am. Coll. Cardiol..

[53] Pitt B., Pfeffer M.A., Assmann S.F., Boineau R., Anand I.S., Claggett B., Clausell N., Desai A.S., Diaz R., Fleg J.L. (2014). Spironolactone for heart failure with preserved ejection fraction. N. Engl. J. Med..

[54] Obadia J.F., Messika-Zeitoun D., Leurent G., Iung B., Bonnet G., Piriou N., Lefèvre T., Piot C., Rouleau F., Carrié D. (2018). Percutaneous Repair or Medical Treatment for Secondary Mitral Regurgitation. N. Engl. J. Med..

[55] COMMITTEE V.-W., Généreux P., Piazza N., Alu M.C., Nazif T., Hahn R.T., Pibarot P., Bax J.J., Leipsic J.A., Blanke P. (2021). Valve Academic Research Consortium 3: Updated endpoint definitions for aortic valve clinical research. Eur. Heart J..

[56] Dweck M.R., Boon N.A., Newby D.E. (2012). Calcific aortic stenosis: A disease of the valve and the myocardium. J. Am. Coll. Cardiol..

[57] Cioffi G., Faggiano P., Vizzardi E., Tarantini L., Cramariuc D., Gerdts E., de Simone G. (2011). Prognostic effect of inappropriately high left ventricular mass in asymptomatic severe aortic stenosis. Heart.

[58] Généreux P., Pibarot P., Redfors B., Mack M.J., Makkar R.R., Jaber W.A., Svensson L.G., Kapadia S., Tuzcu E.M., Thourani V.H. (2017). Staging classification of aortic stenosis based on the extent of cardiac damage. Eur. Heart J..

[59] Genereux P., Cohen D.J., Pibarot P., Redfors B., Bax J.J., Zhao Y., Prince H., Makkar R.R., Kapadia S., Thourani V.H. (2023). Cardiac Damage and Quality of Life After Aortic Valve Replacement in the PARTNER Trials. J. Am. Coll. Cardiol..

[60] Genereux P., Stone G., O’Gara P., Gravel G.M., Redfors B., Giustino G., Pibarot P., Bax J., Bonow R., Leon M. (2016). Early aortic valve replacement versus a conservative strategy for asymptomatic severe aortic stenosis: Meta-analysis of observational studies. J. Am. Coll. Cardiol..

[61] Sá M.P.B.O., Cavalcanti L.R.P., Escorel Neto A.C.A., Perazzo Á.M., Simonato M., Clavel M.-A., Pibarot P., Lima R.C. (2019). Early Aortic Valve Replacement versus Watchful Waiting in Asymptomatic Severe Aortic Stenosis: A Study-Level Meta-Analysis. Struct. Heart.

[62] Ullah W., Gowda S.N., Khan M.S., Sattar Y., Al-Khadra Y., Rashid M., Mohamed M.O., Alkhouli M., Kapadia S., Bagur R. (2020). Early intervention or watchful waiting for asymptomatic severe aortic valve stenosis: A systematic review and meta-analysis. J. Cardiovasc. Med..

[63] Yuan T., Lu Y., Bian C., Cai Z. (2020). Early Aortic Valve Replacement vs. Conservative Management in Asymptomatic Severe Aortic Stenosis Patients With Preserved Ejection Fraction: A Meta-Analysis. Front. Cardiovasc. Med..

[64] Kang D.-H., Park S.-J., Lee S.-A., Lee S., Kim D.-H., Kim H.-K., Yun S.-C., Hong G.-R., Song J.-M., Chung C.-H. (2019). Early Surgery or Conservative Care for Asymptomatic Aortic Stenosis. N. Engl. J. Med..

[65] Banovic M., Putnik S., Penicka M., Doros G., Deja M.A., Kockova R., Kotrc M., Glaveckaite S., Gasparovic H., Pavlovic N. (2022). Aortic Valve Replacement Versus Conservative Treatment in Asymptomatic Severe Aortic Stenosis: The AVATAR Trial. Circulation.

